# Advances in Educational Neuroscience: Current Status and Future Directions

**DOI:** 10.3390/brainsci16070706

**Published:** 2026-06-30

**Authors:** Antonio Luque De La Rosa, Alejandro Vargas Serrano, Celia Gallardo Herrerías

**Affiliations:** Departamento de Educación, Universidad de Almería, Carretera Sacramento s/n, 04120 Almeria, Spain; avs268@ual.es (A.V.S.); cgh188@ual.es (C.G.H.)

Educational neuroscience has established itself over the last few decades as an interdisciplinary field capable of bridging the gap between research on brain function and teaching and learning processes. In a context marked by profound technological, social, and educational transformations, this Special Issue brings together contributions that reflect the methodological, conceptual, and applied diversity that currently characterizes the discipline. The works presented here address fundamental questions related to cognitive development, educational inclusion, the consequences of global crises such as the COVID-19 pandemic, the integration of artificial intelligence into educational processes, and the use of advanced neuroscientific tools to better understand human learning.

One of the central themes of this Special Issue is the study of executive functions as the foundation of learning and academic development. Along these lines, Tsermentseli, Pavlidou, and Kouklari present evidence on the effects of a school-based intervention aimed at children with specific learning disorders. Their results show significant improvements in working memory, planning, cognitive flexibility, and recognition of mental and emotional states, reinforcing the idea that executive functions are skills that can be trained within mainstream educational settings. This work provides valuable evidence of the potential of school-based interventions to promote both cognitive and socio-cognitive development in populations with specific educational needs.

The importance of executive functions is also reflected in the study by Fonseca et al., who analyze the educational consequences of the COVID-19 pandemic on Brazilian students. Their findings reveal a significant impact on inhibitory control, working memory, and various academic competencies, especially in the upper grades of primary education. Beyond the immediate effects of the health crisis, this study highlights the relevance of in-person schooling as a space for cognitive stimulation, social interaction, and addressing educational inequalities.

The issue of educational equity also features prominently in this volume. The review by Sánchez Amate, Luque de la Rosa, and Tadeu examines the situation of students with autism spectrum disorder during the pandemic, highlighting how existing structural barriers intensified during periods of lockdown and remote learning. The authors show that the disruption of specialized support, difficulties accessing technology, and increased family burden generated significant educational and emotional consequences. Their conclusions underscore the need to build more resilient, inclusive, and prepared educational systems to respond to future emergencies.

Another relevant thematic area in this issue focuses on the growing convergence between educational neuroscience and artificial intelligence. The systematic review by Paglialunga and Melogno analyzes the effectiveness of AI-based interventions for students with learning difficulties. Although the available results are promising and point to substantial improvements in different academic domains, the authors caution about the need to strengthen the methodological quality of future research. This work encourages combining enthusiasm for technological innovation with a rigorous evaluation of its effectiveness and potential limitations.

From a broader perspective, the extensive review by Gkintoni and colleagues explores how artificial intelligence, machine learning, and educational neuroscience are redefining traditional teaching and learning models. By integrating cognitive load theory with neurophysiological data obtained through EEG, fNIRS, and other technologies, the authors describe an emerging scenario characterized by adaptive systems capable of personalizing instruction in real time. However, they also emphasize that the future of these tools will depend on their ability to guarantee principles of equity, privacy, transparency, and accessibility.

Methodological innovation is another of the highlights of this Special Issue. The work by Szostakiwskyj and colleagues proposes a novel procedure for analyzing EEG data in ecological contexts, allowing the study of complex cognitive processes when response times vary among participants and situations. This methodological advance represents an important step toward bringing neuroscience research closer to real-world educational settings.

Building on these advances, future research in educational neuroscience should focus on consolidating ecological validity, further developing the use of wearable neuroscience technologies to study the brain within the dynamic social context of the real classroom. Likewise, the imminent convergence of neuroscience and artificial intelligence demands a coordinated effort to design adaptive learning systems that not only demonstrate methodological and empirical efficacy but are also grounded in ethical principles of privacy, transparency, and equity. Finally, it is crucial to promote longitudinal studies that evaluate the long-term impact of cognitive and socio-cognitive interventions, especially in vulnerable populations or those affected by structural inequalities. Only through this interdisciplinary, ethical, and methodologically rigorous approach will it be possible to transform neuroscience findings into pedagogical policies and practices that foster truly inclusive, resilient, and personalized education.

